# A study on age group differences in gender awareness among female employees in the hotel industry

**DOI:** 10.3389/fpsyg.2026.1914591

**Published:** 2026-10-29

**Authors:** Hui Wang, Ziyi Ji, Peilai Wang

**Affiliations:** 1 Shanghai Institute of Tourism, Shanghai Normal University, Shanghai, China; 2 Zhejiang Urban Governance Studies Center, Hangzhou, Zhejiang, China; 3 SILC Business School, Shanghai University, Shanghai, China; 4 School of Hospitality Management and Culinary Arts, Shanghai Institute of Tourism, Shanghai, China

**Keywords:** age group differences, female hotel employees, gender consciousness, hospitality human resource management, mixed methods

## Abstract

**Introduction:**

Women constitute a growing share of the hospitality workforce. Differences in gender consciousness between younger and older female hotel employees remain under-explored, particularly outside Western contexts, as do the factors that account for them.

**Methods:**

This study examined such differences among 302 female hotel employees in China, drawing on an explanatory sequential mixed-methods design in which a structured survey of two age groups (151 aged 18 to 44 and 151 aged 45 and above) was followed by semi-structured interviews of ten employees (five from each age group). Grounded in social role theory and generational cohort theory, the survey examined two related gender-relevant constructs, feminine communal orientation and gender equality awareness, while interview data were used to explore participants’ interpretations of the observed differences.

**Results:**

Younger employees showed significantly higher feminine communal orientation and more egalitarian gender attitudes than middle-aged and older employees in the primary group comparisons. After adjustment for educational attainment, hotel-industry tenure, and job level, the age-group difference in feminine communal orientation was attenuated and no longer statistically significant, whereas the difference in gender equality awareness remained significant. Interview participants attributed these differences to three factors, namely educational attainment and knowledge structure, exposure to media and information technology, and changes in social environment and family structure.

**Discussion:**

Cohort membership in this workforce was closely intertwined with tenure, job level, and educational composition. The findings extend gender consciousness research, previously concentrated in media, education, and general labor market settings, into the hospitality workforce, and they suggest that uniform human resource policies may serve this workforce poorly. Practical implications include transparent promotion criteria, gender-neutral job design, and needs-responsive gender-awareness initiatives aimed at supporting female employees across different career circumstances.

## 1 Introduction

Women now represent close to half of the global hospitality workforce (UN Women World Tourism Organization, 2019), yet this numerical presence has not translated into proportionate access to leadership or equitable career outcomes (Russen et al., 2021). A persistent body of research documents pronounced occupational segregation in the sector: women are disproportionately concentrated in lower-skilled, service-oriented roles, while men are more likely to occupy supervisory and administrative positions, a pattern associated with a measurable gender gap in job quality that widens with age (Santero-Sanchez et al., 2015). This imbalance extends into senior leadership. A 2023 benchmarking report on U. S. and Canadian hotel companies found that, although women have achieved parity with men at the director level, representation declines sharply at more senior levels, with roughly 16 men for every woman at the chief executive level (Jolly et al., 2023). Structural and cultural barriers to women’s advancement into top management positions, including organizational misconceptions about women’s leadership capability and persistent work-life balance constraints, have been documented across multiple national contexts (Segovia-Pérez et al., 2021). Evidence from China further shows that gender inequality in hospitality organizations is reproduced through both workplace discourse (Liu et al., 2022) and evaluations of women in leadership roles (Chun et al., 2024). Women working in hospitality may actively negotiate and challenge hegemonic gender expectations in their everyday employment practices, while implicit and explicit attitudes toward female hospitality managers continue to shape how women’s managerial suitability is evaluated (Liu et al., 2022; Ngan and Litwin, 2022). These organizational processes are embedded in broader Chinese social and economic structures that continue to influence women’s access to employment opportunities, career advancement, and decision-making authority (Yavan, 2025).

A growing body of hospitality scholarship has examined these structural and cultural mechanisms, and a recent systematic review of women’s career development in the hospitality industry explicitly calls for research that situates these barriers within their broader sociocultural context (O’Neill et al., 2025). One dimension of that context that remains under-examined is generational change. The hotel industry has simultaneously undergone marked workforce “youthification,” with younger employees entering the workforce alongside more tenured employees who began their careers under different organizational norms (Goh and Lee, 2018). Prior work specifically examining the intersection of gender with age in hotel careers suggests that privilege and disadvantage accumulate differently across cohorts, shaping who has access to the same nominal career opportunities (Mooney et al., 2017). Central to the present study is the construct of gender consciousness: employees’ own understanding of, and attitudes toward, gender roles and gender equality in the workplace, encompassing how they interpret gendered role assignments, whether they regard prevailing arrangements as fair, and what treatment they expect from their organizations. Yet despite growing attention to generational dynamics in hospitality work, comparatively little research has examined how gender consciousness varies across generational cohorts of female hotel employees, or what accounts for such variation when it exists.

This gap matters because gender consciousness plausibly shapes how employees interpret and respond to the structural inequities documented above: whether they perceive unequal promotion practices as discriminatory, whether they are willing to challenge traditional role assignments, and whether they expect and demand equitable treatment from their employer. If younger and older female hotel employees differ systematically in gender consciousness, organizations that apply uniform human resource policies across age cohorts may be poorly calibrated to either group’s actual expectations and concerns.

China provides a particularly instructive setting in which to examine these questions. The generational cohorts now working side by side in Chinese hotels came of age under sharply different social conditions compressed into a few decades of rapid transformation: older employees entered the workforce when access to higher education was limited, whereas younger employees grew up amid the massive expansion of higher education that began in 1999 (Si, 2022) and the rapid spread of internet access, which has substantially narrowed the gender gap in basic connectivity (Song et al., 2024). China’s family-planning policies further set the younger cohort apart, producing a generation of predominantly only children whose concentrated parental investment has been linked to a heightened sense of individual self-worth and a greater readiness to resist workplace patriarchal pressures (Liu, 2023). National survey evidence confirms that Chinese women’s gender role attitudes have shifted measurably toward more modern profiles across successive cohorts (Chi and Zhou, 2025). The hotel industry brings these divergent cohorts into sustained daily contact, combining a rapidly entering younger workforce with long-tenured middle-aged and older employees (Zhou et al., 2025), making it an organizational context in which generational contrasts in gender consciousness are likely to be both pronounced and consequential.

Against this backdrop, this study addresses three research questions: (1) Do significant differences in gender consciousness exist between younger and older female hotel employees? (2) What factors do female hotel employees perceive as contributing to the observed differences between the two age groups? (3) How might hotel human resource management respond to the needs and workplace concerns identified across these two employee groups?

This study makes two contributions. Theoretically, it extends gender consciousness research, previously concentrated in media, education, and general labor-market settings, into the empirical context of hospitality work and connects this literature to the hospitality management scholarship on occupational segregation and women’s career advancement summarized above. Practically, by comparing survey and interview data across two age-defined groups of female hotel employees and identifying factors associated with the observed differences, the study provides an evidence base for needs-responsive human resource practices that take account of employees’ workplace and career circumstances.

## 2 Literature review

### 2.1 Gender consciousness and tourism research

Gender consciousness refers to an individual’s cognition, understanding, attitudes, and behavioral tendencies regarding gender roles, shaped by social context and personal experience, including education, family environment, occupational history, and exposure to broader cultural and media influences (Tabassum and Nayak, 2021). In work organizations, gender consciousness carries direct behavioral consequences: it informs how employees respond to gendered task allocation, whether they regard advancement and reward systems as legitimate (Khan et al., 2021), and how actively they pursue or forgo career opportunities, tying it to outcomes of central concern to hospitality employers, including satisfaction, commitment, and retention (Liu et al., 2021).

A substantial body of recent empirical research, much of it conducted in the Chinese context, has examined how gender role attitudes form and change, with education receiving the greatest attention (Du et al., 2021; Zhang and Zhu, 2024). The collective message of this literature is one of conditionality rather than uniform effect: additional schooling has been found to promote more egalitarian attitudes under some circumstances (Du et al., 2021), to erode them where educational expansion coincided with a deterioration in women’s relative labor-market position (Si, 2022), and to improve career-related but not marriage-related attitudes where patriarchal family norms persisted (Zhang and Zhu, 2024). Evidence from Chinese workplaces points in the same direction, with higher education attenuating the link between traditional gender attitudes and work–family conflict among employees (Chen et al., 2022). Education, in short, shapes gender consciousness in interaction with the institutional, labor-market, and organizational contexts in which individuals live and work, which directs analytic attention toward the specific occupational settings in which gender consciousness is formed and enacted.

A second strand of research has examined gender role attitudes using mixed-methods designs that combine quantitative measurement with qualitative inquiry into underlying mechanisms, an approach methodologically close to the present study. Chen and Zhang (2024) used a mixed-methods design to examine gender differences in the relationship between gender role attitudes and happiness in China, finding that the association between egalitarian attitudes and well-being differed by gender and highlighting the value of combining survey and qualitative data to interpret these patterns. Relatedly, recent large-scale evidence suggests that Chinese women’s gender role attitudes cluster into distinct types, ranging from multidimensional traditional to fully modern orientations, with the relative sizes of these groups shifting over time toward more modern profiles (Chi and Zhou, 2025). This heterogeneity highlights the value of examining multiple gender-relevant orientations rather than relying on a single undifferentiated indicator. The present study therefore considers two related but conceptually distinct constructs: feminine communal orientation and gender equality awareness. Gender consciousness is likely to be especially salient in the hotel industry, where the gendered organization of work is visible in daily routine. Job allocation remains strongly patterned by sex. Housekeeping and front-of-house service roles are staffed predominantly by women, while maintenance, security, and senior management skew male (Santero-Sanchez et al., 2015; Kokkranikal et al., 2024). Women’s representation also thins toward the top of the hierarchy (Segovia-Pérez et al., 2021), and frontline service work carries sustained emotional and appearance demands that fall disproportionately on female employees. Gender consciousness supplies the frame through which employees interpret these arrangements. It shapes whether gendered task allocation and unequal advancement are experienced as given or as contestable, and whether organizational equality initiatives are received as legitimate or as merely performative (Je et al., 2025). Since these encounters accumulate over a career, and since gendered advantage and disadvantage in hotel work vary with age (Mooney et al., 2017), the industry is also a setting in which differences between age cohorts in gender consciousness are likely to be pronounced.

Research conducted in the Chinese hospitality context demonstrates that gendered employment is not sustained solely through formal occupational segregation. Women employees may actively reinterpret and challenge hegemonic gender expectations, while attitudes toward female hospitality managers remain shaped by both conscious evaluations and implicit gender associations (Liu et al., 2022; Ngan and Litwin, 2022). Viewed within the broader social and economic context of gender inequality in China, these findings suggest that gender consciousness concerns not only abstract beliefs about equality but also how female employees interpret, reproduce, and contest gendered workplace expectations (Yavan, 2025).

Despite this progress, two gaps remain salient for the present research. First, existing studies on gender role attitudes in China have concentrated primarily on general population samples, students, or specific subgroups, with comparatively little attention to service-sector employees as a distinct occupational population. Second, tourism and hospitality research has addressed gender mainly through structural outcomes and female tourism experiences. A systematic review of female-focused tourism research found that the literature has concentrated largely on tourist motivation, travel risk, and sexuality, with comparatively limited attention to employees’ own gender consciousness (Zhang et al., 2024). Although previous studies have examined how women in Chinese hospitality organizations challenge gendered workplace discourse and how female hospitality managers are evaluated, this literature has focused primarily on workplace practices, career barriers, and managerial attitudes rather than generational variation in employees’ gender consciousness (Liu et al., 2022; Ngan and Litwin, 2022). Research has therefore established the gendered character of hospitality employment in China, but has not yet explained whether female employees from different age-defined cohorts interpret these arrangements differently. Taken together, the relative neglect of service-sector employees in gender-attitude research and the limited attention to generational variation in hospitality employees’ gender consciousness constitute the research gap addressed by the present study.

### 2.2 Generational research in tourism

Workplace generational research has grown rapidly across organizational psychology in recent years. A 2022 bibliometric analysis identified a sharp rise in studies on Generation Z as employees between 2009 and 2020, most concentrated in the final years of that period (Benítez-Márquez et al., 2022). Generational research in tourism and hospitality has expanded considerably as well, driven primarily by the entry of Generation Z into the workforce as Baby Boomer and Generation X employees approach retirement. A recent critical synthesis of the empirical literature identified Generation Z as the largest and fastest-growing generational group in the hospitality industry (Seyfi et al., 2024). Its members bring distinct work-related expectations as both customers and employees, including a stronger orientation toward sustainability, transparency, and digitally integrated work environments than prior generations. The same synthesis argued that hospitality organizations have been slow to adapt their workforce practices to these shifting expectations. This generational shift carries direct human resource consequences. In a time-lagged study of 426 Generation Z employees across 20 upscale Chinese hotels, Zhou et al. (2025) found that organizational socialization shaped early retention primarily through its effect on perceived person-environment fit. This effect was further moderated by employees’ career commitment. Conventional onboarding and retention practices designed around older cohorts may therefore not transfer directly to younger employees. Beyond hospitality, generational diversity has also been shown to shape team-level outcomes. In a three-wave survey of multi-generational teams within a Chinese organization, Wang and Duan (2025) found that generational diversity gave rise to both cognitive and affective conflict, which exerted opposing effects on team innovation. Shared leadership amplified the benefits of cognitive conflict but did not buffer the costs of affective conflict. These findings underscore that age group differences shape not only individual attitudes but also team dynamics, and that they require active managerial attention to translate into positive outcomes. Qualitative evidence points in the same direction. A study of hotel directors and human resource managers in Porto, Portugal found that Millennial and Generation Z employees present distinct recruitment and retention challenges relative to older cohorts (Sousa et al., 2026). These challenges include stronger expectations around flexibility, meaningful work, and rapid career progression. The study recommended that hotels adopt generationally differentiated rather than uniform human resource strategies in response.

What remains largely absent from this generational literature, however, is a gender lens. Evidence from adjacent research nonetheless suggests that generational and gender dynamics are closely intertwined. In hotel careers specifically, gendered privilege and disadvantage vary with age, so that women of different generations encounter systematically different opportunity structures (Mooney et al., 2017). Research on women of China’s first one-child generation likewise shows that a distinctly generational sense of self-worth shapes how these women negotiate workplace gender pressures (Liu, 2023). Generational membership, in short, conditions not only what employees expect from work but also how they experience its gendered dimensions.

These strands, however, have yet to converge. The hospitality generational literature has concentrated on generic work attitudes and organizational outcomes such as job satisfaction, person-environment fit, and turnover intention. The evidence connecting generation to gender, meanwhile, comes largely from outside hospitality management or addresses careers rather than consciousness itself. Comparatively little research has examined whether and how generational cohorts of hospitality employees differ in their gender consciousness specifically. This neglect is striking because the cohorts entering and exiting the hospitality workforce over the past two to three decades have done so under markedly different social, educational, and informational conditions. As reviewed in Section 2.1, these are precisely the conditions that shape gender-role beliefs in systematically different ways. This gap is particularly salient for the hotel industry given its predominantly female, multi-generational workforce. Generational scholars have linked differences in work values and career expectations to conditions such as differential exposure to higher education, digital media, and changing family structures (Seyfi et al., 2024; Huyler et al., 2025). These are the same conditions that gender-attitudes researchers have identified as key drivers of gender consciousness formation: educational attainment (Du et al., 2021; Si, 2022; Zhang and Zhu, 2024), exposure to media and information technology (Song et al., 2024), and family structure and change (Liu, 2023). The present study addresses this intersection directly by examining differences in gender awareness between younger and older female hotel employees in China.

A note on cohort terminology is warranted. The studies reviewed above employ Western generational labels such as Baby Boomers, Generation X, Millennials, and Generation Z. The present study instead operationalizes generation as two broad cohorts: younger employees aged 18 to 44, and middle-aged and older employees aged 45 and above. In the Western labeling scheme, the younger cohort spans Millennials and Generation Z, while the older cohort spans Generation X and the Baby Boom generation. Collapsing Millennials and Generation Z into a single younger cohort is theoretically appropriate in the Chinese context. The conditions most relevant to gender consciousness formation divide Chinese cohorts at approximately the 1980 birth-year watershed rather than along Anglo-American label boundaries. Employees born after roughly 1980 came of age amid the post-1999 expansion of higher education (Si, 2022), the rapid diffusion of the internet (Song et al., 2024), and predominantly one-child family structures (Liu, 2023). Those born earlier completed their formative years before these transformations. Consistent with generational cohort theory, which defines cohorts by shared formative conditions rather than by fixed calendar labels (Huyler et al., 2025), the present study therefore treats these two broad cohorts as the theoretically meaningful generational contrast in this setting. However, these groups are treated empirically as age groups in the present analysis, because the cross-sectional design does not permit cohort effects to be separated from age, career stage, or other compositional differences.

### 2.3 Conceptual framework and hypotheses

The two strands of literature reviewed above converge on an unaddressed intersection. Research on gender consciousness has established that education (Du et al., 2021; Si, 2022; Zhang and Zhu, 2024), exposure to media and information technology (Song et al., 2024), and labor-market conditions (Si, 2022) shape women’s gender-role attitudes, yet it has rarely examined service-sector employees as a distinct occupational population. Hospitality research, for its part, has documented age group differences in work values and persistent structural gender inequality, but has not treated employees’ gender consciousness as an outcome in its own right. Studies in the Chinese hospitality context have examined women’s responses to hegemonic gender discourse and attitudes toward female managers, but they have not investigated whether the gender consciousness underlying such responses varies across age-defined workforce groups (Liu et al., 2022; Ngan and Litwin, 2022). To our knowledge, no study has directly examined whether female hospitality employees of different generations differ in gender consciousness, or what accounts for such differences. The closest existing work examines how gender intersects with age in hotel careers (Mooney et al., 2017), but its focus is career privilege and disadvantage rather than gender consciousness itself.

The study’s core constructs are grounded in social role theory. On this account, the historical distribution of women and men into different social roles generates role-based expectations that individuals internalize as gender norms, which then shape self-concept, attitudes, and workplace behavior (Hanek and Garcia, 2022). Gender consciousness, as used here, denotes the internalized interpretive frame that results from this process, determining whether gendered arrangements at work come to be seen as legitimate or as open to challenge. Consistent with evidence that such attitudes are multidimensional rather than a single continuum (Chi and Zhou, 2025). The present study examines two related gender-relevant constructs: feminine communal orientation and gender equality awareness.

Feminine communal orientation refers to the extent to which employees describe themselves in terms of communal attributes traditionally coded as feminine, such as warmth, empathy, sensitivity, and emotional engagement. Social role theory suggests that communal attributes have historically been associated with women’s caregiving and relational roles (Hanek and Garcia, 2022). These attributes are also occupationally salient in hospitality because frontline service frequently requires interpersonal warmth, emotional responsiveness, and relational engagement (Santero-Sanchez et al., 2015; Kokkranikal et al., 2024). In the present study, feminine communal orientation therefore captures employees’ self-reported endorsement of these feminine-coded communal attributes. It does not directly measure employees’ awareness that these attributes are gendered or their identification with a gendered workplace identity.

Gender equality awareness reflects the normative aspect by measuring how much employees agree with traditional, unequal gender roles in areas such as work, family, career advancement and treatment at work. Higher scores suggest stronger adherence to these traditional views, whereas lower scores indicate more egalitarian attitudes, consistent with prior research on gender-role attitudes and equality beliefs (Du et al., 2021; Zhang and Zhu, 2024). The two constructs are conceptually related but distinct. Feminine communal orientation concerns self-reported communal attributes traditionally coded as feminine, whereas gender equality awareness concerns normative beliefs about gender roles and equal treatment.

Generational cohort theory explains why these constructs differ across age groups. People who grow up during the same formative period tend to develop lasting values and perspectives unique to their cohort (Huyler et al., 2025). As described in Section 2.2, the two groups in this study experienced very different circumstances that influenced their attitudes about gender roles, including access to higher education (Si, 2022), exposure to digital media (Song et al., 2024), and family structure (Liu, 2023). For the younger cohort, these factors all move in the same direction. Greater access to education and widespread digital media broaden exposure to ideas about gender equality, while being an only child often means more parental attention for daughters and a greater emphasis on independence. As a result, younger employees are likely to develop a stronger sense of their role at work as it relates to gender, while being less likely to hold traditional gender-role beliefs compared to those whose formative years came before these social changes.

The quantitative phase examines two main hypotheses. First, younger female hotel employees between the ages of 18 and 44 are expected to show a stronger sense of feminine communal orientation compared to their middle-aged and older counterparts, who are 45 and above. Second, younger employees are anticipated to be less likely to endorse traditional gender-role beliefs, as indicated by lower scores on the gender equality awareness dimension, than the older group. The qualitative phase serves an explanatory purpose. It explores the second research question by uncovering the reasons participants believe these differences exist, as revealed by the survey. This approach, where a quantitative phase focused on testing hypotheses is followed by a qualitative phase that seeks to explain the findings, is a hallmark of the explanatory sequential mixed-methods design used in this study (Creswell and Plano Clark, 2018).

## 3 Materials and methods

This study used an explanatory sequential mixed-methods approach, following the steps outlined by Creswell and Plano Clark (2018). It began with a quantitative phase and continued with a qualitative phase to clarify the findings. The first phase involved a structured questionnaire that assessed feminine communal orientation and gender equality awareness, making it possible to compare responses from younger and older female hotel employees. Once the survey data had been collected and analyzed, the second phase began with semi-structured interviews. These conversations focused on how participants view gender today, what they see as the sources of age group differences in gender attitudes, their experiences of intergenerational interactions and conflicts, and their broader thoughts about working life. The goal was to uncover the factors that might explain the age group differences highlighted by the survey.

### 3.1 Survey

Data for this study were collected in Shanghai between December 2024 and March 2025 through convenience sampling, employing both paper and online questionnaires. The online questionnaire was distributed via a publicly shared link posted on the lead researcher’s WeChat Moments and hosted on the Wenjuanxing survey platform. Respondents self-selected in response to this openly shared link rather than being sampled from a predetermined list of participating properties, so the exact number of hotels represented in the online portion of the sample could not be precisely tracked. Offline paper questionnaires were distributed through the lead researcher’s personal contacts within the hospitality industry, including hotel human resources staff and colleagues at other properties. Eligibility required participants to be female and currently employed in the hotel industry, with the sample spanning different regions and job levels and no additional restrictions on tenure or employment type. The target of approximately 150 participants per age group was set with reference to established guidelines for multi-group structural equation modeling, which recommend a minimum of approximately 100 cases per group for stable parameter estimation (Kline, 2005). The study initially targeted 300 questionnaires (150 per age group). A total of 302 valid questionnaires were ultimately obtained (151 aged 18–44 and 151 aged 45 years and above), representing a 100% valid response rate with no questionnaires excluded from the final analytic sample.

The age boundary distinguishing the two cohorts was set at 45 in advance, based on three considerations. From a theoretical perspective, this cutoff reflects the birth-year divide discussed. At the time of data collection, employees aged 45 or older were born before 1980, and thus their formative years occurred prior to the expansion of higher education after 1999, as well as before the widespread adoption of the internet and the transition to predominantly one-child family structures (Si, 2022; Song et al., 2024; Liu, 2023). In contrast, employees aged 18 to 44 experienced these social changes during their formative years. The boundary also separates individuals who were raised entirely in the post-1978 reform era from those whose formative years either predated or overlapped with its beginning. From an institutional standpoint, this threshold has significance in the Chinese labor market. During the study period, the statutory retirement age for female employees was 50 for worker positions and 55 for cadre positions, meaning that employees aged 45 or older were in a distinct late-career stage within the hotel workforce. The final sample included 151 participants in each age group. The age boundary was established conceptually rather than selected *post hoc* to create equal group sizes.

#### 3.1.1 Descriptive statistics

Table 1 summarizes the demographic profile of the two groups. Among younger employees, 56.29% were aged 18–30 and 43.71% were aged 31–44; just over half (51.66%) held a senior high or secondary vocational qualification as their highest level of education, while roughly 30% held an associate’s or bachelor’s degree. About one-third had between 6 months and 1 year of tenure in the hotel industry, and a further 31.79% had between 1 and 3 years; more than 70% occupied front-line positions, and 59.60% held urban household registration.

**Table 1:** Demographic profile of younger and middle-aged/older female hotel employees.

Variable	Category	Younger (n)	Younger (%)	Older (n)	Older (%)
Education	Junior high or below	23	15.23	64	42.38
	Senior high/secondary vocational	78	51.66	73	48.34
	Associate degree	19	12.58	13	8.61
	Bachelor’s degree	24	15.89	1	0.66
	Postgraduate or above	7	4.64	0	0.00
Tenure	< 6 months	38	25.17	0	0.00
	6–12 months	49	32.45	0	0.00
	1–3 years	48	31.79	0	0.00
	3–5 years	13	8.61	31	20.53
	> 5 years	3	1.99	120	79.47
Job level	Intern	38	25.17	0	0.00
	Front-line employee	108	71.52	80	52.98
	Middle management	5	3.31	58	38.41
	Senior management	0	0.00	13	8.61
registration	Rural	61	40.40	45	29.80
	Urban	90	59.60	106	70.20
Total		151	100.0	151	100.0

Younger group: 18–44 years (*n* = 151); middle-aged/older group: 45 years and above (*n* = 151).

Among middle-aged and older employees, 68.21% were aged 45–49, 23.18% were aged 50–54, and 8.61% were 55 or older. Educational attainment was concentrated at the senior-high/secondary-vocational level (48.34%) and junior-high or below (42.38%); only 0.66% held a bachelor’s degree. Nearly 80% had more than 5 years of hotel-industry tenure, reflecting considerably greater occupational stability than the younger cohort. Just over half (52.98%) occupied front-line positions, while a notable share (38.41%) held middle-management roles, and 70.20% held urban registration roles.

Comparing the two groups, both concentrated similarly at the senior high/secondary vocational education level, but younger employees showed markedly higher overall educational attainment, with substantially greater representation at the associate’s degree level and above. Both groups were predominantly composed of front-line employees, though middle-aged and older employees showed considerably greater representation in middle management, consistent with their longer average tenure. Urban registration predominated in both groups. Together, these patterns reflect both the diversity of the hotel workforce and the retention challenges associated with the industry’s recent youthification trend, with clear implications for human resource management.

#### 3.1.2 Measures and reliability

Instead of translating the original English version, this study employed the simplified Chinese adaptation of the Bem Sex Role Inventory (Bem, 1974) as revised by Lu and Su (2003). This revision involved item analysis using Chinese samples, with items exhibiting insufficient discrimination or low factor loadings excluded, thereby enhancing the instrument’s suitability for Chinese respondents. The revised inventory contains 14 masculinity items and 12 femininity items. As the focus of this study is on women’s identification with gendered aspects of service work, the masculinity subscale was omitted. Two items were removed from the femininity subscale, and the remaining 10 items were adapted to the context of hotel service work, maintaining the underlying trait constructs. We use the term feminine communal orientation as a descriptive label for this adapted femininity-item set. The items capture feminine-coded communal attributes rather than employees’ explicit awareness of or identification with a gendered workplace identity. Gender equality awareness was assessed using 10 items measuring attitudes toward gender roles in both public and private domains, including views on household decision-making and the division of labor between men and women. The survey included two 10-item gender-related measures: feminine communal orientation (e.g., perceived warmth, empathy, and emotional engagement at work) and gender equality awareness (e.g., agreement with traditional gender-division statements such as “men should prioritize work outside the home and women the home”, or perceptions of gender neutral treatment in promotion and pay). Items on the gender equality awareness dimension were scored such that higher values indicate greater endorsement of traditional gender role beliefs (i.e., lower gender equality consciousness); this dimension is therefore interpreted as inversely related to egalitarian gender consciousness throughout the results and discussion.

Internal consistency was assessed using Cronbach’s alpha. Overall scale reliability was acceptable for both groups (younger: *α* = 0.746; older: *α* = 0.784), and reliability at the dimension level was high across both subscales and both groups (feminine communal orientation: *α* = 0.926 for younger employees and *α* = 0.929 for older employees; gender equality awareness: *α* = 0.905 for younger employees and *α* = 0.936 for older employees). All corrected item total correlations (CITC) exceeded 0.40, and the removal of any element would not have significantly increased the alpha at the dimensional level. Cronbach’s alpha remains the most widely reported index of internal consistency in psychological research. However, recent methodological commentary cautions that its appropriateness depends on assumptions, such as essential tau-equivalence, that are rarely tested in applied research, and recommends reporting alpha alongside item-level diagnostics, such as CITC values, rather than alpha alone (Zakariya, 2022), a practice followed throughout the present analysis. Table 2 summarizes these reliability statistics. These indices primarily reflect the internal consistency observed within each cohort, rather than ensuring comparability of scores between cohorts.

**Table 2:** Reliability (Cronbach’s alpha) by group and dimension.

Dimension	Items	Younger	Older
Overall scale	20	0.746	0.784
feminine communal orientation	10	0.926	0.929
Gender equality awareness	10	0.905	0.936

All corrected items–total correlations (CITC) > 0.40 for both dimensions in both groups.

#### 3.1.3 Factor structure and measurement invariance

Construct validity was evaluated through content and structural validity. The source instruments from which the items were selected have demonstrated validity in previous studies, thereby providing evidence for the content validity of the item pool. The 20-item composite instrument utilized in the present study had not previously been employed or validated as a single measure. This composite integrates items from two distinct instruments, excludes the masculinity subscale from the original sex-role inventory, removes two femininity items, and adapts the remaining items to align with the context of hotel service work. The factor structure of an adapted set of this kind cannot be assumed in advance, so exploratory factor analysis (EFA) was used to examine whether the intended two-dimensional structure emerged in these data. The analysis was preceded by Bartlett’s test of sphericity and the Kaiser–Meyer–Olkin (KMO) measure of sampling adequacy. For the younger group, KMO = 0.917 and Bartlett’s test was significant (*χ*^2^ = 1689.82, df = 190, *p* < 0.001). For the older group, KMO = 0.945 and Bartlett’s test was likewise significant (*χ*^2^ = 1877.95, df = 190, *p* < 0.001). Both results indicated that the data were well-suited to factor analysis. Factor extraction, retention, and rotation decisions followed current best practice recommendations for EFA, including the use of eigenvalue-based retention criteria and attention to the proportion of variance explained when interpreting the resulting factor structure (Schreiber, 2021).

Principal component analysis with two factors, meeting the eigenvalue > 1 criterion, was performed for both groups. For younger employees, the two factors explained 40.07 and 17.24% of variance, respectively (cumulative 57.31%); for older employees, the two factors explained 42.75 and 19.74% of variance, respectively (cumulative 62.49%). In both groups, the rotated factor solution cleanly separated the feminine communal orientation items (loadings approximately 0.65–0.81) from the gender equality awareness items (loadings approximately 0.67–0.81) onto distinct factors, with no notable cross-loadings, supporting the two-dimensional structure of the scale in this sample.

To further assess whether the two measures operated equivalently across the two age-defined groups, multi-group confirmatory factor analysis (MGCFA) was conducted. A correlated two-factor model was specified, with Items 1–10 representing feminine communal orientation and Items 11–20 representing gender equality awareness. Configural, metric, and scalar invariance were tested sequentially. As shown in Table 3, the configural model demonstrated good fit. Imposing equality constraints on factor loadings produced only negligible changes in model fit, supporting metric invariance. Additional equality constraints on item intercepts likewise did not significantly worsen model fit, supporting scalar invariance across the two groups.

**Table 3:** Multi-group CFA and measurement invariance across age-defined groups.

Panel A. Measurement invariance										
Model	*χ* ^2^	df	CFI	TLI	RMSEA	SRMR	Δ*χ*^2^	Δdf	*p*	ΔCFI
Configural invariance	394.40	338	0.983	0.981	0.024	0.048	–	–	–	–
Metric invariance	417.33	356	0.982	0.981	0.024	0.051	22.93	18	0.193	−0.001
Scalar invariance	432.88	374	0.983	0.982	0.023	0.051	15.55	18	0.624	0.001

Panel B. Latent mean comparisons						
Construct	Younger group	Middle-aged/older group estimate	SE	*z*	*p*	95% CI
Feminine communal orientation	0.000	−0.643	0.102	−6.30	<0.001	[−0.843, −0.443]
Gender equality awareness	0.000	0.752	0.096	7.80	<0.001	[0.563, 0.941]

Younger employees were specified as the reference group, with latent means fixed at zero. Metric invariance constrained factor loadings to equality across groups, and scalar invariance additionally constrained item intercepts. Higher scores on gender equality awareness indicate stronger endorsement of traditional gender-role beliefs and therefore less egalitarian gender attitudes.

Because scalar invariance was established, latent mean comparisons were conducted with the younger group specified as the reference group. The middle-aged/older group showed a significantly lower latent mean for feminine communal orientation and a significantly higher latent mean for gender equality awareness (Table 3). Given that higher scores on gender equality awareness represent stronger endorsement of traditional gender-role beliefs, these results indicate that younger employees expressed more egalitarian gender attitudes. The latent mean comparisons were consistent with the observed-score comparisons reported below.

#### 3.1.4 Correlation, group comparisons, and sensitivity analyses

Pearson correlation coefficients were calculated to examine the relationship between feminine communal orientation and gender equality awareness within each group. In both the younger cohort (*r* = −0.402, *p* < 0.01) and the older cohort (*r* = −0.371, *p* < 0.01), a significant negative correlation was observed. Higher levels of feminine communal orientation corresponded to lower scores on gender equality awareness, indicating less traditional attitudes in both groups. A comparison of the two correlation coefficients using Fisher’s z-test revealed no significant difference (*Z* = −0.31, *p* = 0.75), suggesting that the strength of the association did not vary meaningfully between younger and older employees.

To examine test differences between the two age groups, group membership was recoded (0 = younger, 1 = middle-aged/older; younger = 18–44 years, older = 45 years and above) and independent-samples *t*-tests were conducted on each dimension, with Levene’s test used to assess the equality-of-variance assumption. The primary quantitative objective was to estimate unadjusted differences between the two predefined age cohorts rather than an independent causal effect of cohort membership. Levene’s test was significant for both dimensions (feminine communal orientation: *F* = 12.48, *p* < 0.001; gender equality awareness: *F* = 34.84, *p* < 0.001), so *t*-test results assuming unequal variances are reported. As shown in Table 4, younger employees scored significantly higher on feminine communal orientation (*M* = 3.93, SD = 0.78) than older employees (*M* = 3.31, SD = 0.87), *t*(296.07) = 6.54, *p* < 0.001, mean difference = 0.62, 95% CI [0.44, 0.81]. Younger employees also scored significantly lower on the gender-equality-awareness dimension (*M* = 1.96, SD = 0.70) than older employees (*M* = 2.73, SD = 0.91), *t*(280.08) = −8.31, *p* < 0.001, mean difference = −0.78, 95% CI [−0.96, −0.59], indicating given the reverse-scored nature of this dimension, that younger employees held meaningfully more egalitarian gender attitudes. In this sample, cohort membership was strongly associated with tenure and job level, indicating that observed differences cannot be attributed to cohort effects independently of career stage.

**Table 4:** Independent-samples *t*-test comparing younger and older employees on gender consciousness dimensions.

Dimension	Younger M (SD)	Older M (SD)	*t*	df	*p*	95% CI
Feminine communal orientation	3.93 (0.78)	3.31 (0.87)	6.54	296.07	<0.001	[0.44, 0.81]
Gender equality awareness	1.96 (0.70)	2.73 (0.91)	−8.31	280.08	<0.001	[−0.96, −0.59]

Group coding: younger = 0 (18–44 years, *n* = 151); older = 1 (45 + years, *n* = 151). The mean difference for gender equality awareness is younger minus older, because higher scores on this dimension indicate more traditional attitudes; the negative difference indicates that younger employees held more egalitarian views.

To examine whether the observed age-group differences were sensitive to the substantial compositional differences between the two groups, additional regression analyses were conducted with age group as the focal predictor and educational attainment, hotel-industry tenure, and job level entered as categorical covariates. HC3 robust standard errors were used. Because overlap between the two age groups was limited across several tenure and job-level categories, these models were treated as sensitivity analyses. After adjustment, the age-group difference in feminine communal orientation was attenuated and was no longer statistically significant (*B* = −0.470, SE = 0.277, 95% CI [−1.013, 0.073], *p* = 0.090). In contrast, the adjusted age-group difference in gender equality awareness remained statistically significant (*B* = 0.602, SE = 0.223, 95% CI [0.166, 1.038], *p* = 0.007). Given that higher gender equality awareness scores indicate stronger endorsement of traditional gender-role beliefs, the latter result indicates that the middle-aged/older group continued to report more traditional attitudes after adjustment for these measured compositional characteristics. The sensitivity analysis results are presented in Supplementary Table S1.

### 3.2 Interviews

The qualitative phase explores how participants interpret the observed differences in gender-consciousness scores between the two age-defined groups and what factors they perceive as contributing to these differences.

#### 3.2.1 Interview procedure and participants

Qualitative data were collected through 10 semi-structured interviews (5 younger and 5 middle-aged/older female employees), conducted by telephone or in person, with all participants currently or formerly employed at a single hotel in Shanghai. Participants were invited to provide detailed accounts of their workplace experiences and perspectives. The qualitative sample size was determined in relation to the focused explanatory purpose of the interview phase and the relatively bounded study context. This approach aligns with findings from a recent systematic review (Hennink and Kaiser, 2022), both of which apply to the present investigation. Interview transcripts were analyzed using a team-based thematic analysis. The analysis involved familiarization with the transcripts, initial data-near coding, grouping of related codes into broader categories, and development and refinement of higher-order themes addressing the research questions. Table 5 summarizes participant characteristics; participants are identified only by anonymized codes.

**Table 5:** Interview participant characteristics.

Code	Age	Education	Tenure	Position	Group
P01	24	Bachelor	6 months	Sales intern	Younger
P02	45	Bachelor	23 years	Compensation supervisor	Older
P03	23	Bachelor	8 months	HR intern	Younger
P04	51	Associate degree	12 years	Director of sales & marketing	Older
P05	28	Bachelor	5 years	HR supervisor	Younger
P06	23	Bachelor	6 months	Sales intern	Younger
P07	23	Bachelor	6 months	Switchboard intern	Younger
P08	47	Primary school	2 years	Housekeeping attendant	Older
P09	54	Senior high	10 years	Western restaurant server	Older
P10	60	Senior high	15 years	Stewarding manager	Older

As indicated in Table 4, the age distribution among younger participants was relatively homogeneous, whereas older participants exhibited greater variability in age. All members of the younger cohort possessed a bachelor’s degree. In contrast, educational attainment among older participants ranged from primary school completion to a bachelor’s degree. Tenure and career stability were generally lower within the younger cohort. Notably, only one participant (P05) continued employment at the hotel following completion of an internship and subsequently advanced to a supervisory position.

Among participants in the older cohort, educational attainment and hierarchical position were not correlated. For instance, the most senior participant (P04), who served as director of sales and marketing, possessed an associate degree, while the individual with a bachelor’s degree (P02) occupied a supervisory role, and another participant educated to the senior high school level (P10) held a managerial position. The sample composition does not simply reflect a dichotomy between interns and managers. Two of the five older participants occupied frontline positions (P08, P09), and one of the five younger participants held a supervisory post (P05). Educational composition is theoretically relevant to the cohort contrast because access to higher education changed substantially across the formative periods represented by the two groups. At the same time, education may account for part of the observed between-group difference. The present design cannot determine whether education functions primarily as a cohort-linked mechanism, a compositional covariate, or both. Hierarchical position was not incorporated into the cohort framework, and the interview data do not allow for exclusion of career stage as a contributing factor. For example, participant P08 articulated perspectives aligned with the older cohort, despite not holding a managerial role or having extended tenure, while participant P05 exhibited attitudes characteristic of the younger cohort in conjunction with a supervisory position. Although these instances provide suggestive evidence, the limited sample size precludes definitive conclusions on this matter.

#### 3.2.2 Trustworthiness

The credibility of the study was established through triangulation of quantitative and qualitative research findings. All interviews were conducted by the second author, an undergraduate student specializing in hotel management who is preparing to enter the industry. This background may have influenced the research process in two principal ways. First, it may have facilitated the development of rapport with younger participants in frontline roles or internships, whose professional experiences and career stages closely resembled her own, thereby encouraging more candid responses. Second, as a member of the younger generation, the interviewer may have paid greater attention to narratives reflecting egalitarian perspectives or resonating with her generational cohort. Interview transcripts were returned to participants where appropriate for factual clarification and to identify possible misunderstandings. Analytic transparency was supported through a documented progression from initial codes to broader categories and higher-order themes. The resulting codes and themes are presented in Tables 6, 7, which provide an audit trail from the original transcripts to the final thematic outcomes. Initial coding was conducted by the second author, and the other two authors reviewed the coded material, category boundaries, and developing themes. Differences in interpretation were discussed collectively to refine the analysis. Generalizability is supported by the inclusion of extensive descriptive data through direct quotations. The sample comprised 10 interviews, with five participants in each group. The sample comprised 10 interviews, with five participants in each group. All 11 codes had emerged by the seventh interview, and interviews eight through 10 were reviewed against the existing codebook by all three authors, who independently confirmed that no additional codes were warranted. A new code was defined as a concept or concern raised by a participant that could not be classified under any code already in the codebook. The interview data analysis process is described here to promote transparency and to elucidate its influence on rapport-building with participants and the interpretation of their responses.

**Table 6:** Initial codes and illustrative excerpts.

Illustrative excerpt	Initial code	Category	Code	Function
“The male manager in our hotel seemed to receive more tolerance from senior leadership than my female manager did… his promotion was faster than the other two female managers.” (P03)	Implicit promotion barriers	Workplace gender discrimination	A1	Workplace context
“I try to see if, after talking with my manager and making sure they understand my work, I might also get a chance to be promoted.” (P03)	Proactive strategies in response to inequality	Coping mechanisms	A2	Response (RQ3)
“I’ve noticed our recruiting manager tends to favor better-looking young men over women for front-desk roles.” (P03)	Reverse gender preference in front-of-house hiring	Industry stereotypes	A3	Workplace context
“The younger staff in our department might be around 30; the two more senior people are our banquet sales managers.” (P01); “Most people in our department are in their thirties or forties.” (P06)	Manager cohorts skew younger	Organizational age structure	A4	Workplace context
“Our generation is younger, so our thinking is naturally more current-we like browsing online…” (P05); “Mostly through continuous learning, including recent posts online about unfair treatment of women.” (P07)	Media and information technology shape awareness	Pathways of consciousness formation	A5	Explanatory factor (RQ2)
“My father told me not to worry, that he could still support me-that I could rest if I did not want to work.” (P05)	Parental and family support	Influence of upbringing	A6	Explanatory factor (RQ2)
“There used to be offline training sessions; now it’s [a hotel group’s] online course, offered every year.” (P02)	Limited gender-awareness training	Organizational training gaps	A7	Explanatory factor (RQ2)
“I’m from the countryside and did not have much schooling-I went to work right after junior high.” (P08); “My education is not very high-I finished senior high and did not continue studying.” (P10)	Generational education gap	Educational background differences	A8	Explanatory factor (RQ2)
“I finished senior high and went straight to work-I never felt I was cut out for more schooling, and now I’m about to become a grandmother.” (P09)	Persistence of traditional norms	Traditional-belief differences	A9	Explanatory factor (RQ2)
“I think older female employees may need this kind of training even more… they are just not as sensitive to gender issues as younger women are.” (P07)	Perceived need for gender-awareness training	Training needs	A10	Response (RQ3)
“I think the hotel still needs to provide better benefits for younger women.” (P03)	Calls for stronger protection of women’s rights	Rights-protection demands	A11	Response (RQ3)

Codes A1, A3, and A4 describe the workplace context and are not carried forward into the thematic analysis. Codes A5 to A9 are developed into the three themes presented in Section 4. Codes A2, A10, and A11 inform the practical implications.

**Table 7:** Selective coding: core themes and supporting categories.

Illustrative excerpt	Category	Codes	Theme
“Employees with lower education communicate quite differently from those with higher education.”	Effect of education on gender consciousness	A7, A8	Educational attainment and knowledge structure
“Mostly through continuous learning, including recent online posts about unfair treatment of women.”	Influence of online information on younger women	A5	Media and information technology
“As an only child, my family is relatively simple, and my parents devote all their attention and support to me alone.”	Distinctive family support	A6, A9	Social environment and family change
“If I do not see any prospect of promotion in this department, and I’ve already talked with my manager, I may leave.”	Younger employees’ assertive responses	A2	Coping strategies (individual level)
“I think older women may need this kind of training even more-they are just not as sensitive to gender equality as younger women.”	Gender-awareness training as an organizational response	A10	Coping strategies (organizational level)
“Things like maternity leave and marriage leave should not be cut back.”	Improving welfare provisions	A11	Coping strategies (organizational level)

#### 3.2.3 Coding and theme development

Initial coding of the interview transcripts yielded 11 data-near codes, summarized in Table 5 together with illustrative excerpts. The coding process was conducted inductively, ensuring that the codes closely reflected participants’ expressed concerns. Upon completion, the codes were organized according to three primary functions. The first category consists of three codes that capture the gendered workplace conditions experienced by participants (A1, A3, A4), providing the contextual foundation for the operation of gender consciousness; these codes were not further developed into overarching themes. The second category comprises five codes that participants identified as contributing to age group differences in gender consciousness (A5 to A9). These codes were subsequently integrated into the three themes detailed in Section 4, which address the second research question. The third category includes three codes pertaining to individual and organizational responses to gender inequality (A2, A10, A11), which inform the practical considerations discussed in Section 5.2 in relation to the third research question. The final column of Table 5 specifies the function assigned to each code. The analysis therefore progressed from initial data-near codes to broader categories and, where relevant to the explanatory research questions, to higher-order themes. These codes spanned perceived workplace gender discrimination, individual coping strategies, industry stereotypes, the age structure of management teams, the role of media and information technology in shaping awareness, family support, gaps in organizational gender awareness training, educational differences between age groups, the persistence of traditional gender norms, age group differences in training needs, and demands for stronger protection of women’s workplace rights.

## 4 Factors associated with between-group differences

This study identified three themes related to educational attainment and knowledge structure, exposure to media and information technology, as well as changes in social environment and family dynamics. These themes collectively address the second research question. The 11 initial codes were grouped into broader categories, which were subsequently refined into three principal themes and an additional category concerning individual and organizational responses.

Generational cohort theory conceptualizes these factors as mechanisms that influence the effects of cohort membership. A cohort is characterized by the specific formative conditions experienced by its members, such as educational opportunities, information environments, and family structures (Huyler et al., 2025). The identification of education, media exposure, and family change clarifies how cohort membership may shape gender consciousness. Without these formative conditions, cohort membership would reduce to a simple birth year classification. It is important to note that these factors also exhibit variation within cohorts for reasons unrelated to birth year. For example, differences in educational attainment may arise from urban or rural backgrounds and family resources. Access to digital media can vary according to income level and occupational status, while family circumstances are influenced by marital and parental status. As a result, these factors are only partially associated with cohort membership. The subsequent analysis presents both cohort-related and non-cohort-related patterns as reported by participants.

These three factors demonstrate varying degrees of association with cohort membership. The expansion of higher education after 1999 contributed to higher educational attainment among the younger cohort, indicating a cohort-specific influence. However, educational attainment also varies within each cohort, as discussed in Section 4.1. Exposure to digital media encompasses both formative influences and current access, given that older employees also utilize internet resources in the present context. Changes in family structure reflect both cohort characteristics, such as the prevalence of the one-child policy among younger participants, and life-stage considerations. For instance, the account provided by participant P02 regarding an increased emphasis on job stability following childbirth illustrates caregiving responsibilities that might be similarly described by younger employees in comparable situations. The implications of these distinctions for the present interpretation are examined further in Section 5.3.

### 4.1 Educational attainment and knowledge structure

Participants most frequently identified educational attainment as a key factor influencing their experiences. They consistently associated higher levels of education with an increased emphasis on equal treatment and a stronger focus on fairness in career advancement. The experiences of two participants of similar age illustrate the extent to which educational attainment can function independently of birth cohort. Participant P02, aged 45 and holding a bachelor’s degree, described her transition from the security department—a position she and her family regarded as less suitable for women—into a finance role to pursue promotion opportunities. In contrast, participant P08, aged 47, grew up in a rural environment with limited educational opportunities, worked in factories, and later relocated to Shanghai to be closer to her adult child. Despite their similar ages, their narratives reveal substantial differences, underscoring the independent influence of educational attainment.

Migration experience provides an explanation for divergence observed within the same cohort. Analysis of nationally representative survey data by Yuan and Zhang (2023) demonstrated that individuals migrating from rural to urban areas tended to exhibit greater acceptance of gender equality following migration, especially in relation to the labor market. This attitudinal shift was most pronounced among women originating from regions with more traditional gender norms, and women were more influenced by exposure to urban gender culture than their male counterparts. The experience of participant P08, who combined rural origin, limited educational attainment, and labor migration to Shanghai, illustrates how factors such as migration and education, rather than birth cohort alone, contributed to the formation of her gender consciousness.

This contrast observed within the same cohort challenges a purely generational interpretation in the current study. Educational attainment displays variation not only between cohorts but also within them, as illustrated by these two participants who belong to the same cohort yet differ in educational background, household registration, and occupational trajectory. As a result, education reflects both cohort-related influences, such as the overall increase in attainment following the post-1999 expansion, and factors independent of cohort, including rural or urban origin and family resources. The differences between cohorts described in Section 3.1 may primarily reflect compositional characteristics. Variations in educational composition between the two cohorts could account for differences in gender consciousness, even in the absence of a distinct cohort-specific effect. The implications of this interpretation are further discussed in Section 5.3.

### 4.2 Media and information technology

Three younger interviewees independently referenced the “internet,” “media,” or “film” as important channels shaping their gender consciousness. Younger employees, who have greater access to digital media and information technology, are more readily exposed to messaging about gender equality and women’s rights, which appears to support the development of stronger gender consciousness; older employees, with comparatively less exposure to these channels, appear correspondingly slower to develop such awareness. One participant attributed age group differences in part to the rapid growth of internet access in recent years, noting that more women now encounter gender-related issues through mainstream media, films, and online platforms (P03); another linked her generation’s more contemporary outlook directly to greater time spent online (P05). This pattern is consistent with recent research on Chinese women’s internet engagement, which finds that, despite a substantially narrowed gender gap in basic internet access, persistent disparities remain in how women use digital platforms and the empowerment outcomes they derive from them, with younger, more digitally fluent users better positioned to encounter and engage with online gender equality discourse than less digitally engaged users (Song et al., 2024). The interviews suggest an age-group pattern in digital media exposure among the participants. However, current access to and use of digital media may also vary with education, income, and occupational status. The evidence therefore does not isolate cohort membership as the source of the observed difference.

### 4.3 Social environment and family change

Participants associated the observed differences between younger and older employees with the social and family environments in which they had lived. Some of these accounts referred to formative cohort conditions, whereas others reflected current life stage, career stage, or family responsibilities. Younger employees, raised in a comparatively more open and egalitarian social environment, tend to develop stronger gender consciousness earlier and to display more proactive workplace behavior, actively pursuing fair promotion and compensation and showing greater willingness to challenge perceived gender bias. Older employees, by contrast, often faced greater family and social pressure during their formative years, which may have encouraged more cautious attitudes toward asserting gender related claims, both in their general orientation toward work and in their understanding of gender equality. For example, one younger participant described responding to perceived unfairness vis-à-vis a male peer by working harder to gain recognition from senior leadership, and indicated she would consider leaving the organization if she saw no realistic prospect of advancement after raising the issue with her manager (P03). Older participants articulated perspectives characterized by a greater emphasis on accommodation. For example, one participant indicated that the experience of motherhood increased her appreciation for the stability offered by her current position (P02). Another participant observed that colleagues with longer tenure in her department had, for the most part, ceased to pursue promotion and placed a higher priority on family responsibilities (P06).

These two accounts reflect changes associated with different stages of the life course rather than generational distinctions. The increased importance placed on job stability following parenthood represents an adjustment that typically accompanies family formation, and a younger employee with similar responsibilities might express comparable views. A decrease in promotion-seeking behavior among colleagues with extended tenure likewise corresponds to career stage dynamics. These accounts are presented as reported by participants and are not interpreted as evidence of a cohort effect.

Family structure also changed considerably across the period separating these two cohorts. China’s family planning policies contributed to declining fertility rates and reshaped norms around childbearing. Parental investment in children intensified over the same period, and most employees born after 2000 are only children. Research on women of China’s first one-child generation finds that being the sole focus of parental investment fosters a strong sense of individual self-worth, which these women draw on in negotiating workplace patriarchal pressures (Liu, 2023). Several interviewees described family environments that encouraged independence and discouraged economic dependence on a spouse. One younger participant described her female colleagues as independent women who had moved to Shanghai from elsewhere to build careers on their own, sharing a conviction that women should be economically self-sufficient (P03). Another recounted that when she considered leaving her job but did not want to leave Shanghai, her father reassured her that he could continue to support her financially if needed (P05). These accounts suggest that conditions of upbringing shaped how these participants understood their own autonomy at work. The present findings extend Liu’s account by suggesting that the self-worth associated with this family structure bears on gender consciousness as expressed in workplace settings, beyond the work-family negotiation her study examined.

One aspect of this theme is directly associated with cohort membership in a manner that cannot be attributed to life-course transitions. Family structure at birth represents a fixed condition that cannot be altered retrospectively. Participants in the younger cohort generally grew up as only children, while those in the older cohort did not share this experience. Narratives describing family environments that promoted independence and discouraged economic reliance on a spouse, as reflected in the accounts of participants P03 and P05, pertain to circumstances established during early socialization. An older employee cannot replicate the experience of an only-child upbringing by progressing through different stages of life. Similarly, the social climate experienced during formative years is determined by the historical context in which individuals were raised. The analysis here distinguishes between influences arising from formative conditions and those shaped by current circumstances.

## 5 Discussion

This study examined differences in gender consciousness between younger and older female hotel employees in China. The survey showed that younger employees scored significantly higher on feminine communal orientation and significantly lower on traditional gender-role beliefs than middle-aged and older employees. Interview participants in both age groups described such differences as a familiar feature of the hotel workforce. They attributed them to three factors, namely educational attainment and knowledge structure, exposure to media and information technology, and changes in social environment and family structure. These accounts are consistent with prior research linking gender consciousness to educational attainment, employment status, social interaction, and access to information technology, and they add social environment and family change as further contributing factors. Cohort membership in this workforce was closely intertwined with tenure, job level, and educational composition, so these results describe differences between two groups whose composition differs in several respects at once. The sections below consider what the findings support theoretically, what they suggest for practice, and where the design limits the conclusions that can be drawn.

### 5.1 Theoretical implications

New home economics theory holds that gender-based division of labor within households leads women to weigh family considerations relatively heavily and, at times, to subordinate career advancement to family welfare; market, legal, and cultural forces can compound this pattern by disadvantaging women in the labor market and pushing them out of paid work altogether. Social role theory offers a complementary lens for interpreting the present findings: it holds that perceived differences between women and men arise primarily from their historical distribution into different social roles, with women more often occupying communal, care-giving roles and men more often occupying agentic, leadership roles, and that these role-based expectations become internalized as gender norms that shape individuals’ own self concept and behavior, not merely external constraints imposed on them (Hanek and Garcia, 2022). The primary observed-score comparisons indicated significant differences between the two age-defined groups on both measures, and these patterns were supported by latent mean comparisons after configural, metric, and scalar measurement invariance had been established. However, the subsequent sensitivity analyses qualified the interpretation of these differences. After adjustment for educational attainment, tenure, and job level, the difference in feminine communal orientation was attenuated and no longer statistically significant, suggesting that this pattern may be partly attributable to compositional characteristics of the two groups. By contrast, the age-group difference in gender equality awareness remained statistically significant after adjustment, indicating that the difference in traditional versus egalitarian gender-role attitudes was not fully accounted for by these measured characteristics.

Generational cohort theory provided the conceptual foundation for this study and informed its hypotheses. According to this theoretical framework, individuals who experience similar formative conditions during their development tend to acquire enduring values and perspectives specific to their cohort (Huyler et al., 2025). The observed between-group differences are consistent with the expectations of generational cohort theory, but they should not be interpreted as definitive evidence of an independent cohort effect. Cohort membership was structurally associated with educational attainment, tenure, job level, career stage, and life stage in the present sample. The findings therefore identify differences between two age-defined workforce groups and suggest several plausible formative mechanisms.

A specific aspect of the findings is not fully addressed by alternative explanations. Family structure at birth is determined during formative years and cannot be retrospectively altered. Younger participants predominantly experienced one-child family environments, whereas older participants did not share this background, and no subsequent stage of life can replicate the experience of being raised as an only child. The timing of access to educational opportunities is similarly established early in life, even though the level of attainment may differ within each cohort. It remains an open question whether these factors alone explain the observed differences after accounting for career stage and educational composition.

The theoretical contribution of the study lies partly in demonstrating the methodological difficulty of applying generational cohort theory to workplace populations, where cohort membership, career progression, and life stage are inherently interrelated. In workplace contexts, cohort, career stage, and life stage are inherently interconnected. Employees in older cohorts typically possess greater tenure, have progressed further in their careers, and have reached more advanced stages of family life. Previous organizational research has often applied generational cohort theory without fully addressing the complexity of these overlapping factors. The present findings highlight the necessity of research designs that explicitly account for career stage and life stage when examining cohort effects in workplace populations. Cross-sectional comparisons based solely on age groups are insufficient to disentangle these relationships.

By situating this analysis within the hospitality industry, a context largely absent from prior gender consciousness research, which has concentrated on media, education, and general labor market settings, this study also broadens the empirical foundation of feminist and gender consciousness scholarship.

### 5.2 Practical implications for hotel human resource management

The findings of this study provide hotels with a more rigorous and empirically supported foundation for developing human resource policies. Younger employees demonstrated a heightened aspiration for promotion and were more attuned to disparities in compensation based on gender. In response, hotels may consider enhancing the transparency of promotion processes and formalizing relevant policies. Initiatives such as the pay-equity commitments implemented by Inter Continental Hotels Group or the senior female mentorship programs at Marriott International serve as instructive examples. This recommendation is further supported by evidence from large-scale research on pay transparency. For instance, an extensive study examining pay transparency mandates across multiple jurisdictions found that employer disclosure of salary ranges was associated with a reduction in the gender pay gap by approximately 20–40 percent. This effect was primarily attributed to increased transparency, which limited the capacity of employers to negotiate lower wages with female employees (Baker et al., 2023). These findings imply that analogous transparency measures related to promotion processes may likewise contribute to reducing gender disparities in career advancement within the hotel sector. Related evidence also suggests that perceived organizational unfairness can undermine job satisfaction, while supervisory trust may buffer some of these negative effects, further emphasizing the importance of transparent and trustworthy supervisory processes (Rasheed et al., 2026a,b).

Some hotels, particularly those that are older or smaller in scale, continue to display a gendered division of labor across various roles. In response to this issue, hotels may benefit from implementing gender-neutral job descriptions as a means to mitigate traditional occupational segregation by sex. This challenge is well documented within the hospitality literature. For example, a recent qualitative study examining women’s career advancement in UK hotels found that occupational segregation persisted as a significant structural barrier. Women were often directed into roles such as housekeeping and front of house, which are associated with domestic and feminine skills, while managerial and decision-making positions were disproportionately allocated to men. These patterns remained evident even in organizations with formal diversity policies in place (Kokkranikal et al., 2024). The findings underscore the importance of addressing job design itself, in addition to promotion practices, in order to advance gender equality within the sector. Evidence from another traditionally male-dominated sector similarly shows that organizational justice, person-job fit, and work-life conditions are closely associated with women’s job satisfaction and intention to stay, highlighting the importance of fair and supportive HR practices for female employee retention (Ngah et al., 2026).

Gender-awareness training may form part of a broader organizational response to gender inequality. However, the present study did not directly measure employees’ training needs or evaluate the effectiveness of training interventions, and the interview evidence does not justify prescribing different training solely on the basis of age group. Hotels may instead tailor training to identified employee needs, occupational contexts, and career circumstances. Existing research suggests that the effectiveness of gender-equality initiatives depends on program design and on whether underlying beliefs that justify existing inequalities are adequately addressed (Je et al., 2025).

Beyond formal HR policies, evidence from the pharmaceutical sector indicates that leadership attributes such as delegation and feedback can enhance psychological empowerment and employee engagement, suggesting that supportive supervisory practices constitute another relevant organizational mechanism (Rasheed et al., 2026a).

Collectively, these measures have the potential to enhance women’s motivation and ability to pursue sustained careers in the hotel industry and to contribute to a more gender-equitable workplace.

### 5.3 Limitations and future research

Several limitations should be acknowledged in interpreting these findings. The research was conducted within the hotel industry in Shanghai, and data on hotel category were not collected. As a result, the generalizability of the findings to other sectors or regions requires further investigation. The generational classification in this study included only two broad categories, and future research might benefit from employing more detailed birth-cohort distinctions. Although the present MGCFA supported configural, metric, and scalar invariance across the two age groups, the adapted measures have not yet been independently validated in other hospitality samples. Future research should therefore replicate their factor structure and measurement invariance across broader occupational and cultural contexts.

The two age groups differed substantially in educational attainment, tenure, and job level. To assess the sensitivity of the primary comparisons to these compositional differences, additional regression analyses adjusted for these three characteristics. The age-group difference in feminine communal orientation was attenuated and no longer statistically significant after adjustment, whereas the difference in gender equality awareness remained significant. These findings indicate that measured compositional characteristics account for part of the observed between-group variation but do not fully account for the difference in gender equality awareness. However, the adjusted estimates should still be interpreted cautiously because overlap between the groups was limited across several tenure and occupational categories, residual confounding may remain, and age cannot be separated from birth cohort within a single cross-sectional survey. Accordingly, neither the adjusted nor unadjusted results identify an independent cohort effect. Longitudinal or repeated cross-sectional designs with greater overlap in career-stage characteristics would be required to distinguish cohort-related influences more convincingly from age, life stage, and occupational composition.

The demographic composition of the interview sample is not uniform. All younger participants possessed bachelor’s degrees, and four out of five held intern positions. In contrast, older participants displayed greater variation in educational attainment, ranging from primary school completion to a bachelor’s degree, and occupied positions spanning frontline service to directorial roles. Cohort membership, educational background, and occupational status are closely interrelated within both the interview and survey samples. The number of interviews conducted aligns with established criteria for achieving data saturation in studies with homogeneous and narrowly defined populations. However, each cohort subgroup consisted of only five individuals, which necessitates caution in interpreting generational comparisons based on the interview data.

## 6 Conclusion

This study investigated variations in gender consciousness among younger and older female hotel employees in China by employing an explanatory sequential mixed-methods approach. Survey data were collected from 302 employees and supplemented by semi-structured interviews with 10 participants. The survey results showed that younger employees obtained higher observed scores on feminine communal orientation and lower observed scores on the gender equality awareness dimension, indicating less endorsement of traditional gender-role beliefs, than middle-aged and older employees. The survey results showed significant differences between the two age-defined groups in feminine communal orientation and gender equality awareness. These patterns were further supported by latent mean comparisons after configural, metric, and scalar measurement invariance had been established across the two groups. Sensitivity analyses further showed that the difference in feminine communal orientation was attenuated and no longer statistically significant after adjustment for education, tenure, and job level, whereas the difference in gender equality awareness remained significant. Interview participants associated the observed between-group differences with educational attainment and knowledge structure, exposure to media and information technology, and changes in social environment and family structure. These factors may reflect a combination of cohort-related formative conditions, educational composition, career stage, and life stage.

This study expands the scope of gender consciousness research, which has primarily focused on media, education, and general labor market settings, by examining these dynamics within the hospitality workforce. The findings also highlight a methodological challenge in generational research involving workplace populations. Specifically, cohort effects are closely intertwined with career stage and life stage, making it necessary to employ research designs that carefully measure and account for these overlapping variables in order to isolate the influence of cohort membership.

The findings suggest that hotel human resource management may benefit from needs-responsive and gender-sensitive practices rather than policies based solely on age-group membership. Such practices may include transparent promotion criteria, gender-neutral job design, and gender-awareness initiatives tailored to identified employee needs, occupational contexts, and career circumstances. Future research should replicate the measurement invariance of the adapted measures in independent samples, recruit age groups with greater overlap in education, tenure, and occupational position, and employ longitudinal or repeated cross-sectional designs that allow cohort-related influences to be distinguished more clearly from age, career stage, and life stage.

## Data Availability

The raw data supporting the conclusions of this article will be made available by the authors, without undue reservation.
