# Supplementary material for: A study on age group differences in gender awareness among female employees in the hotel industry
Source: Front Psychol. 2026 Oct 29;17:1914591. doi: 10.3389/fpsyg.2026.1914591 (PMC13755203; doi:10.3389/fpsyg.2026.1914591)
Supplement: Supplementary file 1 [file Table_1.docx]

**Supplementary Table S1**

| **Outcome** | **Unadjusted age-group effect** | **Adjusted age-group effect** | **Robust SE** | **95% CI** | **p** |
| --- | --- | --- | --- | --- | --- |
| FCO | −0.623 | **−0.470** | 0.277 | [−1.013, 0.073] | **0.090** |
| GEA | 0.776 | **0.602** | 0.223 | [0.166, 1.038] | **0.0068** |

0 = younger; 1 = middle-aged/older
